# Age-Related Memory Impairment and Sex-Specific Alterations in Phosphorylation of the Rpt6 Proteasome Subunit and Polyubiquitination in the Basolateral Amygdala and Medial Prefrontal Cortex

**DOI:** 10.3389/fnagi.2021.656944

**Published:** 2021-04-09

**Authors:** Brooke N. Dulka, Sydney Trask, Fred J. Helmstetter

**Affiliations:** Department of Psychology, University of Wisconsin-Milwaukee, Milwaukee, WI, United States

**Keywords:** ubiquitin-proteasome system, trace fear conditioning, aging, amygdala, prefrontal cortex, sex differences

## Abstract

Aging is marked by an accumulation of damaged and modified brain proteins, and the ubiquitin-proteasome system (UPS) is important for cellular protein degradation. Recent work has established a critical role for the UPS in memory and synaptic plasticity, but the role of the UPS in age-related cognitive decline remains poorly understood. We trained young, middle-aged, and aged male and female rats using trace fear conditioning (TFC) to investigate the effects of age and sex on memory. We then measured markers of UPS-related protein degradation (phosphorylation of the Rpt6 proteasome regulatory subunit and K48-linked polyubiquitination) using western blots. We found that aged males, but not aged females, showed behavioral deficits at memory retrieval. Aged males also displayed reduced phosphorylation of the Rpt6 proteasome subunit and accumulation of K48 in the basolateral amygdala, while aged females displayed a similar pattern in the medial prefrontal cortex. These findings suggest that markers of UPS function are differentially affected by age and sex in a brain region-dependent manner. Together these results provide an important step toward understanding the UPS and circuit-level differences in aging males and females.

## Introduction

It is well established that women are at a greater risk for Alzheimer’s disease and age-associated dementias. Although a disparity exists in the representation of female subjects in neuroscience research (Beery and Zucker, 2011), there is also a growing literature that describes genetic and hormonal factors related to age-related memory decline in women (e.g., Frick, 2009; Neu et al., 2017). But, even in the absence of brain disease, the gradual decline of cognitive ability with age is a growing problem among both men and women (Thies and Bleiler, 2013).

Brain aging is marked by an accumulation of damaged and modified proteins in neurons, and this buildup of altered protein is the result of a gradual deterioration of both cellular quality control mechanisms and decreased protein degradation processes (Carrard et al., 2002). The ubiquitin-proteasome system (UPS) is the primary proteolytic mechanism which is responsible for the degradation of these damaged proteins (Saez and Vilchez, 2014). Additionally, dysfunctions in the UPS are associated with neurodegenerative disorders such as Alzheimer’s (Oddo, 2008) and Parkinson’s disease (Betarbet et al., 2005). While a growing body of work highlights the important role of the UPS in regulation of memory and synaptic plasticity in young adult animals (Jarome and Helmstetter, 2013), how altered UPS function contributes to age-related cognitive decline, as well as potential differences between males and females, remains poorly understood.

Trace fear conditioning (TFC) is a simple and well-controlled associative learning procedure that engages many of the same brain circuits required for human episodic memory (Knight et al., 2004; Gilmartin et al., 2014). Task-related neural activity as well as synaptic plasticity linked to TFC memory formation and retrieval are required in the dorsal hippocampus (DH; Yoon and Otto, 2007) and medial prefrontal cortex (mPFC; Gilmartin and Helmstetter, 2010; Reis et al., 2013) as well as in the basolateral region of the amygdala (BLA; Kwapis et al., 2011; Kochli et al., 2015). Successful performance in this memory test is thought to depend on the coordinated interaction of a network of forebrain regions, that are not required when animals are trained with standard “delay” auditory fear conditioning (Esclassan et al., 2009; Gilmartin and Helmstetter, 2010).

We recently demonstrated that impairments in TFC memory in aged male rats are associated with changes in UPS function. Specifically, we found that following memory retrieval 22-mo old male rats had decreased phosphorylation of the Rpt6 proteasome regulatory subunit (pRpt6) and increased accumulation of lysine 48 (K48)-linked polyubiquitinated protein in the BLA (Dulka et al., 2020). The Rpt6 is an adenosine triphosphate (ATP)-dependent regulatory subunit found in the 19S regulatory particle of the proteasome and is phosphorylated at Serine-120 by Ca^2+^ /calmodulin-dependent protein kinase II α (CaMKIIα; Djakovic et al., 2009). Phosphorylation of Rpt6 at Serine-120 is critical for the regulation of increased proteasome activity and activity-dependent changes in synaptic strength (Djakovic et al., 2012). We have previously shown that CaMKII regulates Rpt6 phosphorylation and K48 accumulation during the formation of a long-term fear memory (Jarome et al., 2013), while inhibition of CaMKII prevents retrieval-induced increases in proteasome activity and Rpt6 phosphorylation in the amygdala (Jarome et al., 2016). Both acquisition and retrieval of fear memory also increases UPS-related signaling at amygdala synapses (Jarome et al., 2011; Orsi et al., 2019).

Research suggests that females have lower K48 accumulation in the spinal cord compared to males (Jenkins et al., 2020), and aged female rats may display decreased catalytic activity of the proteasome in the cortex, striatum, and substantia nigra (Zeng et al., 2005). In another study, female fruit-flies show an age-associated increase in basal proteasome expression, while the proteolytic capacity of the three different catalytic subunits of the 20S proteasome complex decline as a function of age in these females. Conversely, males show no change in proteolytic capacity, regardless of age (Pomatto et al., 2017). While these studies highlight interesting differences in basal proteasome expression and catalytic capacity, the effects of learning-related neural activity on UPS markers in the aging female brain remain unknown.

Little has been done to investigate the interaction between age and sex during TFC. Although young females have been shown to learn better than males in trace eye-blink conditioning (Dalla et al., 2009), age was not considered in this study. Other work suggests that there are no differences in TFC memory between young and middle-aged male and female rats (Hodes and Shors, 2007). However, the relationship between old age and sex during TFC has not been explored. Interestingly, some cell signaling pathways in the prefrontal cortex may modulate TFC learning in female, but not in male rats (Kirry et al., 2018), suggesting that this region might show functional difference in TFC based on sex. In the present study, we have sought to extend our previous research by examining the effects of aging on memory decline, phosphorylation of the Rp6 subunit, and K48 accumulation in both males and females across several brain regions critical for TFC memory.

## Materials and Methods

### Animals and Housing Conditions

Subjects were male (*n* = 7 per group) and female (*n* = 10 per group), Fisher 344 (F344) rats obtained from the National Institute on Aging (NIA) colony at Charles River (Raleigh, NC) at the ages of 3-, 15-, and 22-month (mo) old at the time of delivery. The same animals were used for the behavioral and molecular components of this study. The rats were individually housed with *ad libitum* access to water and rat chow. The animal colony was maintained at a 14:10 h light–dark cycle with all experiments occurring under the light portion of the cycle. All experiments were approved by the Institutional Animal Care and Use Committee at the University of Wisconsin-Milwaukee and conducted within the ethical guidelines of the National Institutes of Health (NIH).

### Conditioning Apparatus

Fear conditioning was conducted in a set of four Plexiglas and stainless-steel chambers within sound-attenuating boxes as described previously (Dulka et al., 2020; Trask et al., 2020). Briefly, the floor included 18 stainless steel bars connected to a shock generator (Coulbourn Instruments). Each chamber had a speaker to allow delivery of white noise cues, overhead illumination with a 7.5 W bulb, and ventilation fans to provide a constant background noise (55 dB). The chambers were cleaned with 5% ammonium hydroxide solution between sets of rats (Context A). A set of similar chambers (Context B) served as a shifted context for auditory CS testing. There were several distinct features associated with Context B, including textured Plexiglas flooring, infrared illumination, and 5% acetic acid cleaning solution.

### Trace Fear Conditioning Procedures

All animals were handled for 3 days prior to behavioral manipulation. This consisted of transport to the behavior room and then gentle restraint in a towel. The TFC training was conducted in Context A, while auditory CS testing (retrieval) was conducted in Context B. All animals were trained in TFC on Day 1 with six CS-UCS pairings after a six min baseline (BL). The CS was a 10 s white noise cue (72 dB) and the UCS was a 1 s electric footshock (1 mA). The CS and UCS were separated by a 20 s trace interval (TI), and CS-UCS pairings were separated by a variable intertrial interval (ITI) of 240 s ± 20 s. One day following conditioning, rats received a long-term memory test consisting of two 30 s CS presentations following a 2 min BL period. The two CSs were separated by an ITI of 175 s, and the second CS was followed by a 2 min post-CS period. The ITI and post-CS period were combined into a single stimulus free period (SFP). The SFP timepoint at testing was chosen for analysis because it represented CS-induced fear to the trace interval and, thus, was a time at which the TFC-trained animal would have received the footshock during training (Kwapis et al., 2011; Dulka et al., 2020).

### Behavioral Scoring

Freezing behavior was defined as the cessation of all movement excluding respiration (Fanselow, 1980) and was automatically scored in real-time with FreezeScan 1.0 detection software (Clever Sys, Inc.) calibrated to a trained human observer. Rearing behavior within the conditioning apparatus during the retrieval session was also scored. A rear was defined as when the rat rose on its hind legs (front paws either in the air or using the walls as support) and the position was held for a least 2 s. The rears were scored manually from video by an observer blind to group assignments.

### Crude Synaptosomal Membrane Fractionation

Animals were sacrificed with an overdose of isoflurane 90 min following memory retrieval testing. Brains were rapidly removed and flash frozen on dry ice. We chose a 90 min timepoint to remain consistent with the procedures used in Dulka et al. (2020). Using a cryostat (Leica Biosystems), the BLA, mPFC, and CA1 region of the DH were dissected from the brains. Synaptosomal membrane fractions were obtained using methods described previously (Dulka et al., 2020). Briefly, tissue samples were homogenized in TEVP buffer with 320 mM sucrose and centrifuged at 1,000× *g* for 10 min at 4°C. The supernatant was collected and spun at 10,000× *g* for 10 min at 4°C. The resulting pellet containing the synaptosomal fraction was resuspended in phospho-homogenization buffer (50 mM Tris-HCl, 6 mM sodium deoxycholate, 150 mM NaCl, 1mM NaF, two mini EDTA-free complete protease inhibitor tablets (Roche), 0.1% SDS, 1 mM sodium orthovanadate) and measured using a DC Protein Assay (Bio-Rad).

### Western Blot Method

Following the preparation of the synaptic fractions, protein levels were normalized and were loaded into a 7.5% SDS/PAGE gel and then transferred to PVDF membranes using a Turbo Transfer System (BioRad). Membranes were incubated in 5% milk in Tris-buffered saline (TBS) + 0.1% Tween-20 (blocking buffer) for 1–2 h before being incubated in primary antibody solutions for pRpt6 (ProSci, 1:850), total Rpt6 (tRpt6; Abcam:ab178681; 1:1,000), K48 polyubiquitination (Cell Signaling: #8081, 1:500), or β-actin (Cell Signaling: #3700, 1:1,000) and 3% bovine serum albumen in TBS + 0.1% Tween-20 overnight at 4°C. Membranes were then incubated in the appropriate secondary antibody (1:20,000) in blocking buffer for 60 min at RT (β-actin), 90 min at RT (pRpt6), or 4 h at 4°C (tRpt6; K48). Following a final wash, membranes were prepped in a chemiluminescence solution (Clarity ECL, Bio-Rad) for 3 min. Images were captured and densitometry performed using NIH Genesys. The phosphorylated Rpt6-Serine120 rabbit polyclonal antibody was generated commercially (ProSci) against a synthetic peptide (NH2-CALRND(pS)YTLHK-OH) as described previously (Djakovic et al., 2012; Jarome et al., 2013; Dulka et al., 2020).

### Data Analysis

A three-way analysis of variance (ANOVA) test was used to compare freezing during training and retrieval. These 2 (Sex: male, female) × 3 (Age: 3-mo, 15-mo, 22-mo) × 2 (Period/training: BL, TI; Period/retrieval: CS, SFP) ANOVAs were followed by planned comparisons to explore the effect of age and sex. The rearing behavior was analyzed using a two-way ANOVA followed by Fisher’s LSD *post hoc* tests. For the analysis of protein levels as measured using western blots, one-way ANOVAs followed by Fisher’s LSD *post hoc* tests were used to compare mean optical density across the age groups within each sex. Western blot samples are expressed as a percentage of TFC-trained 3-mo old animals within each sex. To remain consistent with our previous work, pRpt6 and K48 are not taken as a proportion of tRpt6 and β-actin, respectively (Dulka et al., 2020). However, we verified that these control proteins did not differ between groups as a function of age. Additionally, while animals from each age group were represented on each blot, the different sexes were run on different blots. Thus, across sex comparisons were not made for western blot data. Statistical outliers were screened according to the methods outlined in Field (2005). These outliers included two samples in the BLA for pRpt6 (one 15-mo male and one 22-mo male); two samples in the mPFC for pRpt6 (one 15-mo female and one 22-mo female); and 3 samples in the DH for pRpt6 (one 15-mo male, one 15-mo female, and one 22-mo female), as well as one sample in the DH for K48 (one 15-mo male). Alpha was set at 0.05. Data are presented as mean ± standard error of the mean (SEM).

## Results

**Figure 1:**
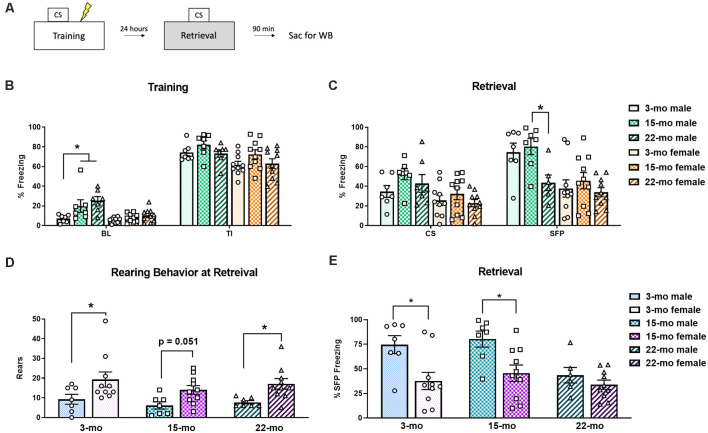
Subjects were trained in trace fear conditioning (TFC) and tested for memory retention 24 h later. **(A)** A schematic of the experimental design. **(B)** During training, both 22 month (mo) and 15-mo males showed more freezing behavior than 3-mo males at baseline (BL). However, all animals reacted normally to shock, as seen by increased freezing during the trace intervals (TI). **(C)** During retrieval testing, while no effects of age were noted during the conditioned stimulus (CS) period, during stimulus free period (SFP) 22-mo old males displayed a deficit in memory retention. No effect of age was noted in females. **(D)** To better understand the behavioral topography of fear responses, rearing behavior was also analyzed during the retrieval test. Overall, females engaged in more rears than males did. **(E)** We also performed planned comparisons across sex within each age group during retrieval testing. At the SFP period, 3- and 15-mo males froze more than their female counterparts, while 22-mo old males did not. Data are shown as mean ± standard error of the mean (SEM). *Indicates *P* < 0.05.

A schematic of the experimental design can be seen in Figure 1A. On the first day, TFC memory acquisition occurred (Figure 1B). A 3-way ANOVA conducted on freezing behavior during this training session found main effects of period (BL vs. TI), *F*_(1, 45)_ = 777.52, *P* < 0.001, sex, *F*_(1, 45)_ = 21.28, *P* < 0.01, and age, *F*_(2, 45)_ = 5.12, *P* = 0.01, and a period × age interaction, *F*_(2, 45)_ = 3.87, *P* = 0.03. No other interactions were significant, largest *F* = 1.29. Planned comparisons showed that during BL, 15-mo males froze more than 3-mo males (*P* = 0.01), while 22-mo males also froze more than 3-mo males (*P* < 0.001). No differences were noted between females at BL (smallest *P* = 0.20). During the averaged TI periods of training, there were no differences among ages or between sexes (smallest *P* = 0.07), suggesting that all the rats acquired the freezing response regardless of age or sex.

On the second day, memory retention was tested (Figure 1C). For this test session, a 3-way ANOVA was conducted to assess behavioral performance. We again found main effects of period (CS vs. SFP), *F*_(1, 45)_ = 36.35, *P* < 0.001 and sex, *F*_(1, 45)_ = 20.97, *P* < 0.001. While there was nearly an effect of age, it did not reach statistical significance, *F*_(2, 45)_ = 3.09, *P* = 0.055. The same was true of the period × age interaction, *F*_(2, 45)_ = 2.48, *P* = 0.095. The three-way interaction (period × sex × age) was also not significant, *F*_(2, 45)_ = 2.13, *P* = 0.13. During the CS timepoint, planned comparisons found that although 3-mo old males tended to freeze less than 15-mo males (*P* = 0.06). Females showed no differences in freezing based on age (smallest *P* = 0.22). This was not completely unexpected, given that we previously found no effect of age in males during the CS (Dulka et al., 2020). It has been suggested that a more appropriate time point to assess trace fear recall is SFP time point because this is when the animal should have learned to expect a shock and, thus, is a conditional response to the auditory cue (Gilmartin et al., 2012). During the SFP time point, 22-mo males froze less than 15-mo males (*P* = 0.02) and tended to freeze less than 3-mo males (*P* = 0.065). Females, on the other hand, showed no differences in freezing (smallest *P* = 0.27).

Planned comparisons within each age group across sexes were also performed to further understand these behavioral effects. During training, we found that at the BL period 15-mo males froze more than 15-mo females, *F*_(1, 45)_ = 8.17, *P* = 0.006, and 22-mo males froze more than 22-mo females, *F*_(1, 45)_ = 12.97, *P* = 0.001. Additionally, during the TI period 3-mo males tended to display higher rates of freezing than 3-mo females, *F*_(1, 45)_ = 4.00, *P* = 0.051. No other planned comparisons at training were significant (largest *F* = 2.54, *P* = 0.12). Differences within each age group across sex were also noted during memory retrieval. During the CS period, while 3-mo males did not differ from 3-mo females (*F* = 1.13), 15-mo males froze more than 15-mo females, *F*_(1, 45)_, = 5.60, *P* = 0.02). During the SFP period, 3-mo males froze more than 3-mo females, *F*_(1, 45)_ = 9.97, *P* = 0.003, 15-mo males froze more than 15-mo females, *F*_(1, 45)_ = 8.77, *P* = 0.005, but 22-mo old animals did not differ from one another, *F*_(1, 45)_ = 2.06, *P* = 0.16 (Figure 1E).

To further understand the influence of sex during the retrieval test, we examined other behaviors as male and female rodents may differ in their behavioral topography in response to conditioned fear (e.g., Gruene et al., 2015a). It was noted during real time testing that some animals spent more time engaging in behaviors such as rearing rather than freezing. Analysis of rearing frequency from video during the retrieval session revealed a significant main effect of sex, *F*_(1, 45)_ = 16.20, *P* < 0.001, such that females engaged in a greater number of rears compared to males overall (Figure 1D). However, there was no main effect of age, *F*_(2, 45)_ = 1.17, *P* = 0.32, and no age × sex interaction (*F* < 1). Planned comparisons show that 3-mo females engaged in more rearing than 3-mo males (*P* = 0.01), 15-mo females tended to rear more than 15-mo males (*P* = 0.051), and 22-mo females also engaged in more rears than their male counterparts (*P* = 0.02). Thus, this increase in rearing might explain the overall pattern of reduced freezing observed during retrieval in females (Figure 1E). Overall, these results replicate our previous finding that aged (22-mo) males have deficits in the retention of a trace fear memory. However, contrary to our expectations, aged females did not display a significant memory deficit.

**Figure 2:**
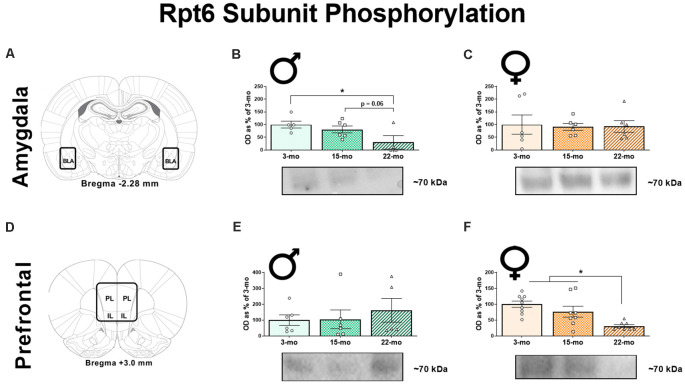
Ninety minutes following the retrieval session, synaptic fractions were obtained in order to analyze the phosphorylation of the Rpt6 subunit using western blots. In the BLA **(A)**, 22-mo old males display reduced pRpt6 **(B)**, while females show no difference **(C)**. However, in the mPFC **(D)**, while males show no differences in pRpt6 **(E)**, females 22-mo old females display reduced pRpt6 **(F)**. Results are expressed as mean percentages of 3-mo old rats within each sex (± SEM). Blots have been cropped for clarity to show representative samples. Here, the mPFC is defined as the area including both the prelimbic cortex (PL) and infralimbic cortex (IL). *Indicates *P* < 0.05.

To determine how age affects memory-driven protein degradation, we next quantified four protein targets of interest (pRpt6, tRpt6, K48, and β-actin) within brain regions known to be critical for TFC. We first quantified pRpt6 and tRpt6 protein in BLA (Figure 2A) synaptic fractions. Replicating our previous work (Dulka et al., 2020), we found that age was associated with a decline in pRpt6 in males, *F*_(2, 12)_ = 3.91, *P* = 0.049 (Figure 2B). Specifically, 22-mo males displayed lower levels of pRpt6 compared to 3-mo males (*P* = 0.02) and 15-mo males (*P* = 0.06). However, no differences in Rpt6 phosphorylation were observed in females (Figure 2C; *F* < 1) in the BLA. Levels of tRpt6 protein did not differ across age groups in males, *F* < 1, or females, *F*_(2, 20)_ = 1.16, *P* = 0.33, suggesting that differences observed in retrieval associated Rpt6 phosphorylation were not due to differences in total Rpt6 protein (Supplementary Figures 1A,B).

In the mPFC (Figure 2D), as in our previous study (Dulka et al., 2020), we found that males displayed no differences in pRpt6 within synaptic fractions, *F*_(2, 14)_ = 0.35, *P* = 0.70 (Figure 2E). However, females showed clear differences based on age, *F*_(2, 22)_ = 9.08, *P* = 0.001 (Figure 2F). Twenty-two month females displayed reduced pRpt6 compared to both 3-mo (*P* < 0.001) and 15-mo old females (*P* = 0.01). Again, tRpt6 levels did not differ in this structure across age groups in males, *F*_(2, 13)_ = 1.08, *P* = 0.37, or females, *F* < 1 (Supplementary Figures 1E,F). Together, these data show that aged males display decreases in Rpt6 phosphorylation in the BLA, while aged females show this pattern in the mPFC.

**Figure 3:**
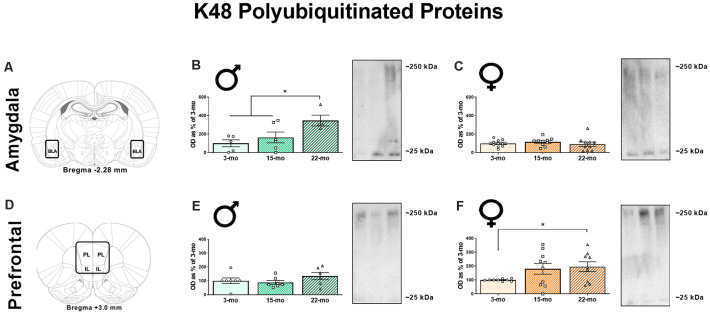
Synaptic fractions were also used to analyze the accumulation of K48-linked polyubiquitination using western blots. In the BLA **(A)**, 22-mo old males display increased K48 **(B)**, while females show no such accumulation **(C)**. However, in the mPFC **(D)**, males show no differences in K48 **(E)**, but females 22-mo old females display increased accumulation of K48 **(F)**. Results are expressed as mean percentages of 3-mo old rats within each sex (± SEM). Blots have been cropped for clarity to show representative samples. The mPFC is defined as the area including both the prelimbic cortex (PL) and infralimbic cortex (IL). *Indicates *P* < 0.05.

We also performed western blots for a lysine 48 (K48) polyubiquitin tag that targets proteins for degradation by the proteasome (Jarome et al., 2013, 2016). In the BLA (Figure 3A), age-related differences in K48 accumulation were observed in males (Figure 3B), *F*_(2, 12)_ = 4.98, *P* = 0.03, but not in females, *F* < 1 (Figure 3C). Specifically, 22-mo males displayed greater K48 levels compared to 3-mo males (*P* = 0.009) and 15-mo males (*P* = 0.04), who did not differ from each other (*P* = 0.40). We also analyzed β-actin in the BLA, and this loading control protein did not differ across age groups in males, *F*_(2, 12)_ = 2.74, *P* = 0.10, or females, *F* < 1, again demonstrating that this effect was specific to proteins associated with memory retrieval (Supplementary Figures 1C,D).

In the mPFC (Figure 3D), males displayed no effect of age on K48 levels, *F*_(2, 17)_ = 1.32, *P* = 0.29 (Figure 3E), but we observed a marginal effect suggesting an accumulation of K48-tagged proteins in females, *F*_(2, 25)_ = 2.64, *P* = 0.09 (Figure 3F). Because of this pattern, we investigated differences across age groups in the females using LSD post-hoc analyses and found that 22-mo old females displayed increased K48 compared to 3-mo old females (*P* = 0.04). Fifteen-mo old females also had increased K48 expression compared to the 3-mo old females (*P* = 0.08). As before, no differences were noted in β-actin levels as a function of age in males, *F*_(2, 18)_ = 1.27, *P* = 0.30, or females, *F*_(2, 25)_ = 2.58, *P* = 0.10 (Supplementary Figures 1G,H). These data show that aged males show increases in K48 accumulation in the BLA, while aged females show increases in K48 in the mPFC.

Although the DH has a longstanding role in trace fear memory, we previously observed no effect of age of the phosphorylation of Rpt6 following TFC in this region (Dulka et al., 2020). However, because this prior study was done solely with male subjects, here we quantified proteins in the DH to see if this pattern was similar or different in females. Consistent with our recent work, no effects on pRpt6 were noted in the DH in males or females, *F*s < 1 (Supplementary Figure 2A). Similarly, no changes in tRpt6 were noted in males, *F*_(2, 14)_ = 1.90, *P* = 0.19, or females, *F* < 1 (Supplementary Figure 2B). No differences were observed in K48 accumulation in males, *F*_(2, 16)_ = 2.55, *P* = 0.11, or females, *F*_(2, 21)_ = 1.86, *P* = 0.18 (Supplementary Figure 2D). Finally, no changes in β-actin were observed in males, *F* < 1, or females, *F*_(2, 25)_ = 1.87, *P* = 0.18 (Supplementary Figure 2C). This suggests that memory-related changes in Rpt6 phosphorylation and K48 accumulation occur in the BLA of males and the mPFC of females.

## Discussion

In the present study, we have found that aged (22-mo old) males displayed a clear deficit in trace fear memory retention similar to what has been reported previously (Moyer and Brown, 2006). Memory deficits in 22-mo old males were associated with decreased phosphorylation of the Rpt6 subunit and a corresponding increase in the accumulation of K48 polyubiquitinated proteins within the BLA (as in Dulka et al., 2020). Alternately, aging in females was associated with changes in the activity-driven markers of UPS activity within the mPFC but not BLA, an opposite pattern to their male counterparts. We found that 22-mo old females displayed clear reductions in pRpt6 and increased accumulation of K48-tagged proteins within the mPFC relative to young adults. These results provide the first evidence that changes in proxy measures for UPS activity such as phosphorylation of the Rpt6 subunit and K48-linked polyubiquitination with aging are reflected in different brain regions in males and females. This may ultimately have important implications for understanding age-related cognitive decline in men and women.

One important consideration here is that pRpt6 and K48 were measured after memory testing, and therefore we cannot separate potential baseline differences in protein degradation between males and females from activity driven by memory retrieval and expression of behavioral responses. Previous work from our laboratory indicates that memory retrieval (compared to a no retrieval condition) specifically promotes Rpt6 phosphorylation (Jarome et al., 2016). While we did not measure phosphorylation of Rpt6 in unstimulated animals, we found here as in prior work (Dulka et al., 2020) that total Rpt6 protein levels did not differ between groups suggesting that the differences we observed, were due to activity driven by memory retrieval. We also cannot rule out the possibility that the memory impairment observed in the aged males during testing was not a result of impaired acquisition.

Another caveat to the present work is that, we only analyzed one cellular component, the synaptic fraction. There may be additional effects of age and sex following TFC memory retrieval in cytoplasmic and nuclear fractions (e.g., Orsi et al., 2019). One recent study found that young male, but not female, rats had increased protein degradation in the nuclei of amygdala cells after fear conditioning, while females had elevated baseline levels of overall UPS activity in amygdala nuclei (Devulapalli et al., 2021). Additionally, we only took brain tissue at 90 min following retrieval. It is possible that females display a different time course of protein expression, as this pattern of expression is currently unknown.

Based on its importance for TFC, one might have expected the DH to show changes in UPS activity following the retrieval of a trace fear memory. This could also be attributable to the choice of cellular compartment analyzed. Prior work has also established that in some situations protein degradation is critical for plasticity in DH (e.g., Lee et al., 2008; Cullen et al., 2017), but the lack of DH effects seen here is consistent with our previous studies that focused on aged males (Dulka et al., 2020; Trask et al., 2020).

Prior work supports age-related deficits in TFC in males, but upto this point female rats have not been included for comparison. We predicted a parallel memory deficit in both sexes as a function of age but did not find significant differences in behavioral performance between old and young females. This lack of effect may represent differential sensitivity of the procedure based on sex, but this seems unlikely given that young animals of both sexes should learn comparably (Kirry et al., 2019). Alternatively, we may not have seen a significant deficit in the 22-mo females due to the large amount of variability in the younger female subjects. While the behavioral outcomes related to sex are to some degree ambiguous, the present study presents compelling data that markers of the UPS are differentially affected by age and sex in brain region-dependent manner.

Recently, there has been growing interest in understanding sex differences in memory networks critical for fear learning (Tronson and Keiser, 2019). Important sex differences may exist throughout the brain, including at the level of neural circuits. For instance, human structural imaging connectivity data suggest that male brains are optimized for intra-hemispheric communication while female brains are optimized for inter-hemispheric communication (Ingalhalikar et al., 2014). In another study in rats, differences in dendritic arborization of infralimbic cortex (IL)-to-BLA neurons between high freezing and low freezing males, but not females, were observed following the retrieval of an extinction memory (Gruene et al., 2015b). Combined with the results of the present study, these data provide evidence that, when examining differences in learning and plasticity between males and females, the effects may be determined in part by the brain region studied. Critical differences in circuit level activity may emerge throughout the lifespan. Because of this, how and why male and female brain circuit activity differs as a function of age is an area of neuroscience research that warrants further investigation.

In sum, the present study is the first to investigate the relationship between aging, sex, trace fear memory, and UPS mechanisms. We show here that age-related memory decline in male rats is associated with decreases in activity-driven phosphorylation of the Rpt6 subunit of the proteasome and increases in accumulation of K48 polyubiquitinated proteins within the BLA. Although females show no significant effect of age on memory retention, they display age-related decreases in pRpt6 and increases in K48 within the mPFC. Altogether these data extend our knowledge of sex differences in aging and protein degradation and provide a compelling first step towards the study of circuit-level differences in aging males and females.

## Data Availability Statement

The raw data supporting the conclusions of this article will be made available by the authors, without undue reservation.

## Ethics Statement

The animal study was reviewed and approved by Institutional Animal Care and Use Committee at the University of Wisconsin-Milwaukee.

## Author Contributions

BD and FH designed the experiments. BD and ST conducted the experiments. BD and ST analyzed the data. BD wrote the manuscript with input from ST and FH. All authors edited, reviewed, and approved the manuscript for publication. All authors contributed to the article and approved the submitted version.

## Conflict of Interest

The authors declare that the research was conducted in the absence of any commercial or financial relationships that could be construed as a potential conflict of interest.

## Supplementary Material

SUPPLEMENTARY FIGURE 1 Control proteins were analyzed using western blots. In the BLA, total Rpt6 (tRpt6) did not differ in males **(A)** or in females **(B)**. β-actin levels also did not differ in males **(C)** or females **(D)**. In the DH, tRpt6 did not differ in males **(E)** or in females **(F)**. Again, β-actin levels also did not differ in males **(G)** or females **(H)**. Results are presented as mean ± SEM.

SUPPLEMENTARY FIGURE 2 Synaptic fractions were also obtained from the DH, and western blots were used to analyze pRpt6, tRpt6, β-actin, and K48-linked polyubiquitination. In males, no differences were observed in pRpt6 **(A)**, tRpt6 **(B)**, β-actin **(C)**, or K48 **(D)**. Similarly, in females, no differences were observed in pRpt6 **(E)**, tRpt6 **(F)**, β-actin **(G)**, or K48 **(H)**. Results are presented as mean ± SEM.

## References

[B1] Beery A. K. Zucker I. (2011). Sex bias in neuroscience and biomedical research. Neurosci. Biobehav. Rev. 35, 565–572. 10.1016/j.neubiorev.2010.07.002 20620164 PMC3008499

[B2] Betarbet R. Sherer T. B. Greenamyre J. T. (2005). Ubiquitin-proteasome system and Parkinson’s diseases. Exp. Neurol. 191, S17–S27. 10.1016/j.expneurol.2004.08.021 15629758

[B3] Carrard G. Bulteau A. L. Petropoulos I. Friguet B. (2002). Impairment of proteasome structure and function in aging. Int. J. Biochem. Cell Biol. 34, 1461–1474. 10.1016/s1357-2725(02)00085-7 12200039

[B4] Cullen P. K. Ferrara N. C. Pullins S. E. Helmstetter F. J. (2017). Context memory formation requires activity-dependent protein degradation in the hippocampus. Learn. Mem. 24, 589–596. 10.1101/lm.045443.117 29038220 PMC5647928

[B5] Dalla C. Papachristos E. B. Whetstone A. S. Shors T. J. (2009). Female rats learn trace memories better than male rats and consequently retain a greater proportion of new neurons in their hippocampi. Proc. Natl. Acad. Sci. 106, 2927–2932. 10.1073/pnas.0809650106 19188598 PMC2650367

[B6] Devulapalli R. Jones N. Farrell K. Musaus M. Kugler H. McFadden T.. (2021). Males and females differ in the regulation and engagement of, but not requirement for, protein degradation in the amygdala during fear memory formation. Neurobiol. Learn. Mem. 180:107404. 10.1016/j.nlm.2021.107404 33609735 PMC8076082

[B7] Djakovic S. N. Marquez-Lona E. M. Jakawich S. K. Wright R. Chu C. Sutton M. A.. (2012). Phosphorylation of Rpt6 regulates synaptic strength in hippocampal neurons. J. Neurosci. 32, 5126–5131. 10.1523/JNEUROSCI.4427-11.2012 22496558 PMC3348785

[B8] Djakovic S. N. Schwarz L. A. Barylko B. DeMartino G. N. Patrick G. N. (2009). Regulation of the proteasome by neuronal activity and CAMKII. J. Biol. Chem. 109:21956. 10.1074/jbc.M109.021956 19638347 PMC2785353

[B9] Dulka B. N. Pullins S. E. Cullen P. K. Moyer J. R. jr. Helmstetter F. J. (2020). Age-Related Memory Deficits are Associated with Changes in Protein Degradation in Brain Regions Critical for Trace Fear Conditioning. Neurobiol. Aging 91, 160–166. 10.1016/j.neurobiolaging.2020.03.001 32280031 PMC7232789

[B10] Esclassan F. Coutureau E. Di Scala G. Marchand A. R. (2009). Differential contribution of dorsal and ventral hippocampus to trace and delay fear conditioning. Hippocampus 19, 33–44. 10.1002/hipo.20473 18683846

[B11] Fanselow M. S. (1980). Conditional and unconditional components of post-shock freezing. Pav. J. Biol. Sci. 15, 177–182. 10.1007/BF03001163 7208128

[B12] Field A. (2005). Discovering Statistics Using SPSS. 2nd Edn. Thousand Oaks, CA, USA: Sage Publications, Inc.

[B13] Frick K. M. (2009). Estrogens and age-related memory decline in rodents: what have we learned and where do we go from here? Horm. Behav. 55, 2–23. 10.1016/j.yhbeh.2008.08.015 18835561 PMC2664384

[B15] Gilmartin M. R. Balderston N. L. Helmstetter F. J. (2014). Prefrontal cortical regulation of fear learning. Trends Neurosci. 37, 455–464. 10.1016/j.tins.2014.05.004 24929864 PMC4119830

[B14] Gilmartin M. R. Helmstetter F. J. (2010). Trace and contextual fear conditioning require neural activity and NMDA receptor-dependent transmission in the medial prefrontal cortex. Learn. Mem. 17, 289–296. 10.1101/lm.1597410 20504949 PMC2884289

[B16] Gilmartin M. R. Kwapis J. L. Helmstetter F. J. (2012). Trace and contextual fear conditioning are impaired following unilateral microinjection of muscimol in the ventral hippocampus or amygdala, but not the medial prefrontal cortex. Neurobiol. Learn. Mem. 97, 452–464. 10.1016/j.nicl.2021.102615 22469748 PMC3358523

[B17] Gruene T. M. Flick K. Stefano A. Shea S. D. Shansky R. M. (2015a). Sexually divergent expression of active and passive conditioned fear responses in rats. Elife 4:e11352. 10.7554/eLife.11352 26568307 PMC4709260

[B18] Gruene T. M. Roberts E. Thomas V. Ronzio A. Shansky R. M. (2015b). Sex-specific neuroanatomical correlates of fear expression in prefrontal-amygdala circuits. Biol. Psychiatry 78, 186–193. 10.1016/j.biopsych.2014.11.014 25579850 PMC4449316

[B19] Hodes G. E. Shors T. J. (2007). Learning during middle age: a resistance to stress? Neurobiol. Aging 28, 1783–1788. 10.1016/j.neurobiolaging.2006.07.012 16971024 PMC3422864

[B20] Ingalhalikar M. Smith A. Parker D. Satterthwaite T. D. Elliott M. A. Ruparel K.. (2014). Sex differences in the structural connectome of the human brain. Proc. Natl. Acad. Sci. 111, 823–828. 10.1073/pnas.1316909110 24297904 PMC3896179

[B22] Jarome T. J. Ferrara N. C. Kwapis J. L. Helmstetter F. J. (2016). CaMKII regulates proteasome phosphorylation and activity and promotes memory destabilization following retrieval. Neurobiol. Learn. Mem. 128, 103–109. 10.1016/j.nlm.2016.01.001 26779588 PMC4754128

[B21] Jarome T. J. Helmstetter F. J. (2013). The ubiquitin-proteasome system as a critical regulator of synaptic plasticity and long-term memory formation. Neurobiol. Learn. Mem. 105, 107–116. 10.1016/j.nlm.2013.03.009 23623827 PMC3786694

[B23] Jarome T. J. Kwapis J. L. Ruenzel W. Helmstetter F. J. (2013). CaMKII, but not protein kinase a, regulates Rpt6 phosphorylation and proteasome activity during the formation of long-term memories. Front. Behav. Neurosci. 7:115. 10.3389/fnbeh.2013.00115 24009566 PMC3757295

[B24] Jarome T. J. Werner C. T. Kwapis J. L. Helmstetter F. J. (2011). Activity dependent protein degradation is critical for the formation and stability of fear memory in the amygdala. PLoS One 6:e24349. 10.1371/journal.pone.0024349 21961035 PMC3178530

[B25] Jenkins E. C. Shah N. Gomez M. Casalena G. Zhao D. Kenny T. C.. (2020). Proteasome mapping reveals sexual dimorphism in tissue-specific sensitivity to protein aggregations. EMBO Rep. 21:e48978. 10.15252/embr.201948978 32090465 PMC7132179

[B26] Kirry A. J. Durigan D. J. Twining R. C. Gilmartin M. R. (2019). Estrous cycle stage gates sex differences in prefrontal muscarinic control of fear memory formation. Neurobiol. Learn. Mem. 161, 26–36. 10.1016/j.nlm.2019.03.001 30851433

[B27] Kirry A. J. Herbst M. R. Poirier S. E. Maskeri M. M. Rothwell A. C. Twining R. C.. (2018). Pituitary adenylate cyclase-activating polypeptide (PACAP) signaling in the prefrontal cortex modulates cued fear learning, but not spatial working memory, in female rats. Neuropharmacology 133, 145–154. 10.1016/j.neuropharm.2018.01.010 29353055

[B28] Knight D. C. Cheng D. T. Smith C. N. Stein E. A. Helmstetter F. J. (2004). Neural substrates mediating human delay and trace fear conditioning. J. Neurosci. 24, 218–228. 10.1523/JNEUROSCI.0433-03.2004 14715954 PMC6729570

[B29] Kochli D. E. Thompson E. C. Fricke E. A. Postle A. F. Quinn J. J. (2015). The amygdala is critical for trace, delay and contextual fear conditioning. Learn. Mem. 22, 92–100. 10.1101/lm.034918.114 25593295 PMC4341367

[B30] Kwapis J. L. Jarome T. J. Schiff J. C. Helmstetter F. J. (2011). Memory consolidation in both trace and delay fear conditioning is disrupted by intra-amygdala infusion of the protein synthesis inhibitor anisomycin. Learn. Mem. 18, 728–732. 10.1101/lm.023945.111 22028394 PMC3207254

[B31] Lee S. H. Choi J. H. Lee N. Lee H. R. Kim J. I. Yu N. K.. (2008). Synaptic protein degradation underlies destabilization of retrieved fear memory. Science 319, 1253–1256. 10.1126/science.1150541 18258863

[B32] Moyer J. R. Jr. Brown T. H. (2006). Impaired trace and contextual fear conditioning in aged rats. Behav. Neurosci. 120:612. 10.1037/0735-7044.120.3.612 16768613

[B33] Neu S. C. Pa J. Kukull W. Beekly D. Kuzma A. Gangadharan P.. (2017). Apolipoprotein e genotype and sex risk factors for Alzheimer disease: a meta-analysis. JAMA Neurol. 74, 1178–1189. 10.1001/jamaneurol.2017.2188 28846757 PMC5759346

[B34] Oddo S. (2008). The ubiquitin-proteasome system in Alzheimer’s disease. J. Cell. Mol. Med. 12, 363–373. 10.1111/j.1582-4934.2008.00276.x 18266959 PMC3822529

[B35] Orsi S. A. Devulapalli R. K. Nelsen J. L. McFadden T. Surineni R. Jarome T. J.. (2019). Distinct subcellular changes in proteasome activity and linkage-specific protein polyubiquitination in the amygdala during the consolidation and reconsolidation of a fear memory. Neurobiol. Learn. Mem. 157, 1–11. 10.1016/j.nlm.2018.11.012 30458285

[B36] Pomatto L. C. D. Wong S. Carney C. Shen B. Tower J. Davies K. J. A.. (2017). The age-and sex-specific decline of the 20s proteasome and the Nrf2/CncC signal transduction pathway in adaption and resistance to oxidative stress in *Drosophila melanogaster*. Aging (Albany NY) 9:1153. 10.18632/aging.101218 28373600 PMC5425120

[B37] Reis D. S. Jarome T. J. Helmstetter F. J. (2013). Memory formation for trace fear conditioning requires ubiquitin-proteasome mediated protein degradation in the prefrontal cortex. Front. Behav. Neurosci. 7:150. 10.3389/fnbeh.2013.00150 24167477 PMC3805936

[B38] Saez I. Vilchez D. (2014). The mechanistic links between proteasome activity, aging and age related diseases. Curr. Genomics 15, 38–51. 10.2174/138920291501140306113344 24653662 PMC3958958

[B39] Thies W. Bleiler L. (2013). 2013 Alzheimer’s disease facts and figures. Alzheimers Dement. 9, 208–245. 10.1016/j.jalz.2013.02.003 23507120

[B40] Trask S. Dulka B. N. Helmstetter F. J. (2020). Age-related memory impairment is associated with increased zif268 protein accumulation and decreased Rpt6 phosphorylation. Int. J. Mol. Sci. 21:5352. 10.3390/ijms21155352 32731408 PMC7432048

[B41] Tronson N. C. Keiser A. A. (2019). A dynamic memory systems framework for sex differences in fear memory. Trends Neurosci. 42, 680–692. 10.1016/j.tins.2019.07.009 31473031

[B42] Yoon T. Otto T. (2007). Differential contributions of dorsal vs. ventral hippocampus to auditory trace fear conditioning. Neurobiol. Learn. Mem. 87, 464–475. 10.1016/j.nlm.2006.12.006 17251041

[B43] Zeng B. Y. Medhurst A. D. Jackson M. Rose S. Jenner P. (2005). Proteasomal activity in brain differs between species and brain regions and changes with age. Mech. Ageing Dev. 126, 760–766. 10.1016/j.mad.2005.01.008 15888331

