# Supplementary figures and images for: Age-Related Memory Impairment and Sex-Specific Alterations in Phosphorylation of the Rpt6 Proteasome Subunit and Polyubiquitination in the Basolateral Amygdala and Medial Prefrontal Cortex
Source: Front Aging Neurosci. 2021 Apr 9;13:656944. doi: 10.3389/fnagi.2021.656944 (PMC8062735; doi:10.3389/fnagi.2021.656944)

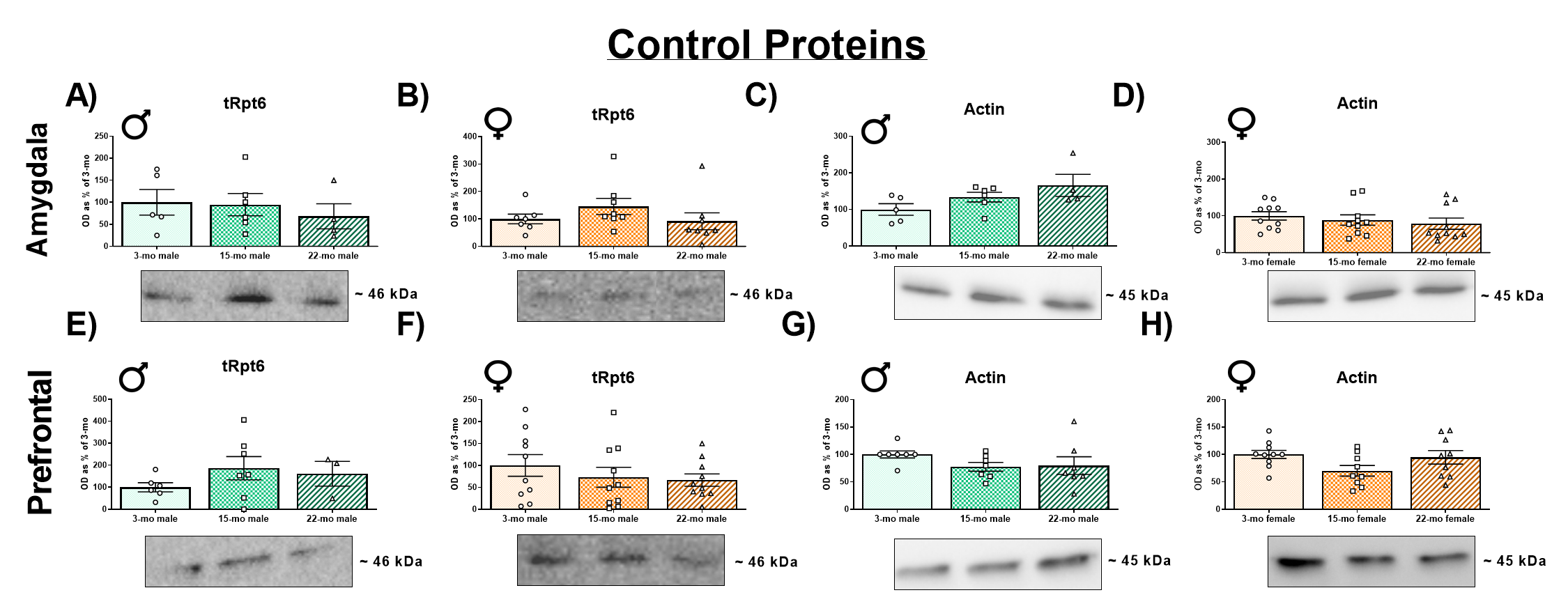

Supplement: SUPPLEMENTARY FIGURE 1 — Control proteins were analyzed using western blots. In the BLA, total Rpt6 (tRpt6) did not differ in males (A) or in females (B). β-actin levels also did not differ in males (C) or females (D). In the DH, tRpt6 did not differ in males (E) or in females (F). Again, β-actin levels also did not differ in males (G) or females (H). Results are presented as mean ± SEM. [file Image_1.TIF]

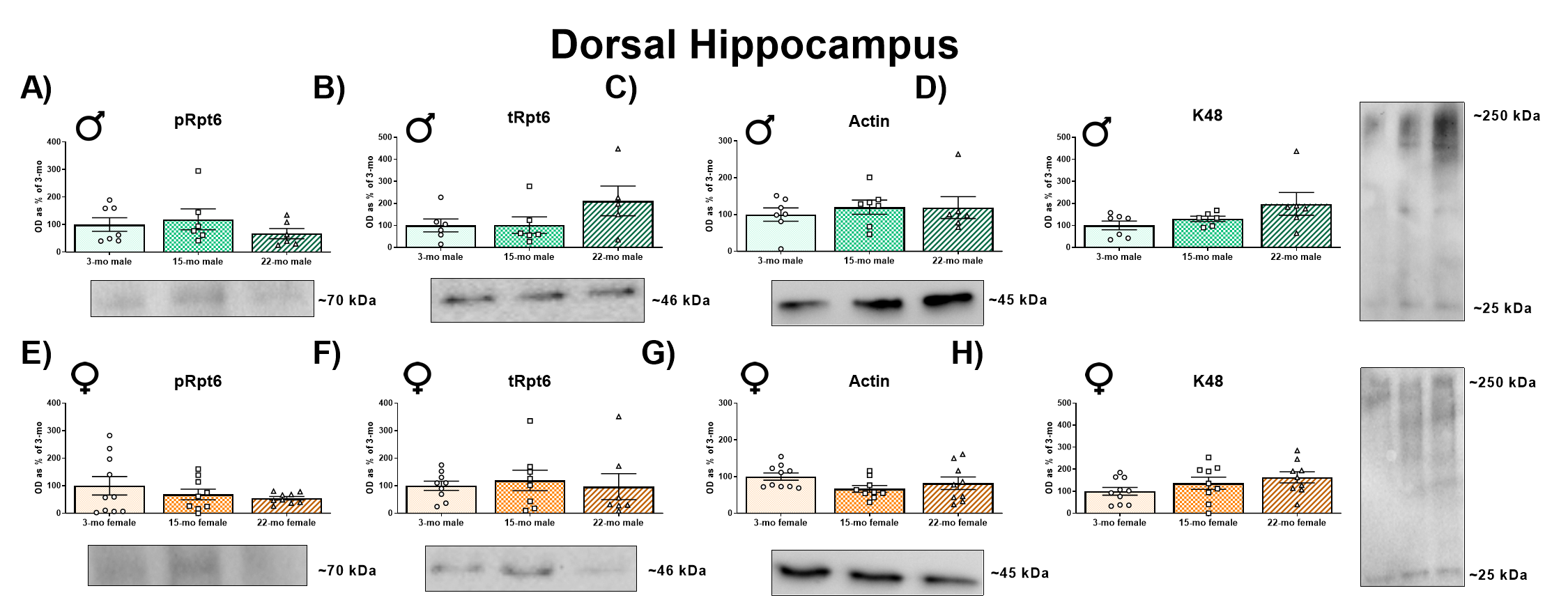

Supplement: SUPPLEMENTARY FIGURE 2 — Synaptic fractions were also obtained from the DH, and western blots were used to analyze pRpt6, tRpt6, β-actin, and K48-linked polyubiquitination. In males, no differences were observed in pRpt6 (A), tRpt6 (B), β-actin (C), or K48 (D). Similarly, in females, no differences were observed in pRpt6 (E), tRpt6 (F), β-actin (G), or K48 (H). Results are presented as mean ± SEM. [file Image_2.TIF]
